# Time course of conflict processing modulated by brief meditation training

**DOI:** 10.3389/fpsyg.2015.00911

**Published:** 2015-07-03

**Authors:** Yaxin Fan, Yi-Yuan Tang, Rongxiang Tang, Michael I. Posner

**Affiliations:** ^1^Dalian Blood Center, Dalian, China; ^2^Department of Psychological Sciences, Texas Tech University, Lubbock, TX, USA; ^3^Department of Psychology, University of Oregon, Eugene, OR, USA; ^4^Department of Psychology, Washington University in St. Louis, St. Louis, MO, USA

**Keywords:** IBMT, conflict resolution, self-control, brain state, ACC, N2, P3

## Abstract

Resolving conflict is a pivotal self-control ability for human adaptation and survival. Although some studies reported meditation may affect conflict resolution, the neural mechanisms are poorly understood. We conducted a fully randomized 5 h trial of one form of mindfulness meditation—integrative body-mind training (IBMT) in comparison to a relaxation training control. During the Stroop word-color task, IBMT group produced faster resolution of conflict, a smaller N2 and an earlier and larger P3 component of the event-related brain potentials. These results indicate that brief meditation training induces a brain state that improves the resolution of conflict.

## Introduction

Our previous randomized studies have shown that integrative body-mind training (IBMT) improves attention and self-regulation after short-term practice (Tang et al., 2007; Tang and Posner, 2009, 2014). Furthermore, 5 days of IBMT training can induce better conflict resolution in the flanker and Stroop task than same amount of relaxation training (RT; Tang et al., 2007; Fan et al., 2014). IBMT is one form of mindfulness meditation and originates from Eastern contemplative tradition and seems to work by increasing brain activity of self-regulation areas such as the anterior cingulate cortex (ACC) and improving its connection to the parasympathetic branch of the autonomic nervous system (Tang et al., 2009, 2012a; see more on Training Methods).

The Stroop effect has often been used as the gold standard for assessing conflict resolution (Stroop, 1935; MacLeod, 1991). The Stroop interference effect (or conflict resolution) refers to the longer time that it takes to name the ink color of a color-word when the ink color and printed color-word are incongruent (e.g., “RED” in blue ink, meaning the “conflict condition”) as compared to congruent (e.g., “RED” in red). Long-term meditation training has been found to improve the efficiency of the executive attentional network measured by the paper Stroop task (Chan and Woollacott, 2007). Wenk-Sormaz (2005) showed that meditation practice led to a reduction in habitual patterns of response on the Stroop task, however, another study failed to find the same effects of mindfulness meditation (Anderson et al., 2007). Compared to a waiting list control, meditators showed better performance on Stroop task (Moore and Malinowski, 2009; Moore et al., 2012; Teper and Inzlicht, 2013); however, these studies were not randomized and could not provide conclusion on causality (Moore and Malinowski, 2009).

In an event-related potentials (ERPs) study of the manual Stroop task, P3 is defined as the largest positive peak following the N1-P2-N2 complex within a latency window between 280 and 600 ms (Ilan and Polich, 1999). P3 timing provides a measure of stimulus classification time that is independent of response processes. P3 amplitude reflects the amounts of attentional resources employed in a given task (Polich, 2007). Stroop interference occurs only after initial color and word processing and is most closely related to the response selection stage (Ilan and Polich, 1999; Rosenfeld and Skogsberg, 2006).

In a long-term meditator group with 2.5–40 years of experience, P3 amplitude was reduced for distracting stimuli in the oddball task, suggesting that meditators had a different attention allocation than non-meditators (Cahn and Polich, 2009). Studies have shown that the fronto-parietal N2 reflects conflict-related activity in the ACC (Van Veen and Carter, 2002; Rueda et al., 2004, 2005). Compared to a waiting list control, meditation training also influences N2 and P2 components, but the direction of these results are inconsistent (Moore et al., 2012; van Leeuwen et al., 2012). Taken together, ERP components can be modulated by meditative practice, although whether these findings are due to training or a pre-existing property of those who choose meditation remains unclear.

In the present study, we aimed to use ERPs to extend our previous behavioral findings and examine the time course of the neural correlates of processing conflict using a standard computerized Stroop task in a randomized design with 5 h of IBMT training. Prior to and following training, all subjects performed the standard Stroop word-color task while their brain activity was measured using ERPs. We hypothesized that (1) after 5 h training, IBMT group will have more reduced Stroop interference effect in reaction time than for relaxation group (2) IBMT will alter the N2 and P3 Stroop-related ERP components through the ACC. These new results will advance our understanding of brain mechanisms of conflict processing related to mental training.

## Materials and Methods

### Subjects and Task

Thirty-five healthy undergraduates (mean age = 21.31, 17 male) without any prior meditation or RT experience participated in this study. All subjects were right-handed, and had normal or corrected-to-normal vision. The experiment was approved by a local Institutional Review Board, and informed consent was obtained from each participant. The experiment consisted of neural, congruent, and incongruent stimuli programmed by E-prime (Psychology Software Tools, Sharpsburg, PA, USA). The congruent stimuli consisted of the three color words in Chinese (red, yellow, blue) written in the same color in which the stimulus was presented (e.g., the word red written in red color). The incongruent stimuli consisted of the same three words with display colors that do not match the word meaning (e.g., the word yellow written in red color). Each incongruent stimulus appeared in either of the two colors that does not match its meaning. In the neutral condition, two no-color words (ball, watch) were presented in one of the three colors.

Subjects were instructed to rest their right middle three fingers on the left three keys of the E-prime response box, and each finger represents one color. They were told that a gray cross would always appear first in the center of the screen serving as a fixation point, and then one word written in different colors would appear. The order is as follows: the fixation point appeared for 600 ms, the word appeared for 150 ms, and then the empty screen appeared for 1950 ms. Subjects were asked to identify the color in which the stimulus was written as fast and accurately as possible and responded by pressing the button of the corresponding color. The experiment was divided into a practice phase and a test phase. The test phase was about 3 min. The formal test consisted of three blocks of 90 trials (30 congruent stimuli, 30 neutral stimuli, 30 incongruent stimuli), each block was around 15 min. Participants were instructed to avoid blinking and eye movement of any sort and to keep their eyes fixated on the monitor rather than looking down at their fingers during task performance. Participants rested briefly after finishing one block. Before and after training, all subjects performed a Stroop word-color task while their brain activity was measured using a high-density electroencephalography system.

### Training Methods

Integrative body-mind training involves body relaxation, mental imagery and mindfulness training, accompanied by selected music background. Cooperation between the body and the mind is emphasized in facilitating and achieving a meditative state (Tang et al., 2007, 2012b). The trainees concentrated on achieving a balanced state of body and mind guided by an IBMT coach. The method stresses no effort to control thoughts, but instead a brain state of restful alertness that allows a high degree of awareness of body, mind, and external instructions. RT involves the relaxing of different muscle groups over the face, head, shoulders, arms, legs, chest, back, and abdomen, guided by a qualified tutor. With eyes closed and in a sequential pattern, one is forced to concentrate on the sensation of relaxation, such as the feelings of warmth and heaviness. This progressive training helps the participant achieve a physical and mental relaxation and calmness (Tang et al., 2007, 2012b). Eighteen subjects had 10 consecutive IBMT sessions with about 30 min per day (5 h in total), 17 subjects were given the same amount of RT. These two training sessions were conducted in parallel.

### Electrophysiological Recording and Analysis

Brain electrical activity was recorded using a 61-channel EasyCap with sintered Ag/AgCI electrodes (Brain Products system). The reference electrode was placed on the FCz, while ground was linked to the AFz. Signals were collected at 500 Hz samples and impedances kept below 5 kΩ. Vertical and horizontal electro-oculograms (EOG) were recorded by electrodes situated supra-and infra-orbital of the left eye and external canthi of both eyes, respectively.

The EEG was digitally low-pass filtered at 20 Hz, and transformed to an average reference. Trials with EOG artifacts (exceeding ± 70 μV), and those contaminated by other artifacts (amplifier clipping or peak-to-peak deflection exceeding 100 μV) were excluded before averaging. The averaged epoch for ERP was 1200 ms, and the first 200 ms before stimulus presentation served as baseline. Only segments with correct responses were averaged, and at least 52 trials were available for each subject and condition (incongruent, congruent, and neural).

Grand averages were computed to identify components and time windows for statistical analysis. Based on the ERPs grand averaged map and the Stroop-related scalp regions (Liotti et al., 2000; Qiu et al., 2006), the following three electrode points were chosen for statistical analysis: Fz, FCz, and Cz. The P2, N2, and P3 peak amplitudes, which were detected from individual ERPs, were measured within time windows of 150–250 ms, 250–400 ms, and 300–500 ms, respectively. The peak amplitude and peak latency data of each component were compared between two groups using between-group, repeated measure ANOVA with Greenhouse–Geisser correction. The significance level for all statistical tests was *p* < 0.05, marginally level was *p* < 0.08.

To map the brain location of ERPs, we used source analysis toolbox, the standardized low-resolution brain electromagnetic tomography software (sLORETA; Pascual-Marqui, 2002). Previous research showed that sLORETA can model and correctly localize the cortical sources of the P3 component at the ACC (BA 24, BA 32) and mesial temporal lobes (Pascual-Marqui et al., 2002; Lorenzo-López et al., 2008).

## Results

Using reaction time and accuracy as dependent variables, we conducted a repeated measured ANOVAs comparing Group (IBMT and Relaxation), Session (Pre and Post) and Congruency (Congruent, Incongruent, and Neutral). The reaction time analysis revealed significant main effects of Session [*F*(1,33) = 48.42, *p* < 0.001] and Congruency [*F*(2,66) = 69.36, *p* < 0.001], as well as significant interactions for Session × Group [*F*(1,33) = 15.38, *p* < 0.001] and Session × Congruency [*F*(2,66) = 7.17, *p* < 0.01]. A main effect for congruency [*F*(2,66) = 47.97, *p* < 0.001] was the only significant result obtained for accuracy.

*Post hoc* analyses indicated that compared to the pre-training scores, both IBMT and relaxation groups showed significant reduction in the post-training reaction time for congruent, incongruent and neutral conditions (all *p* < 0.01). Conflict scores refer to the difference between congruent and incongruent conditions. The pre vs. post difference in conflict reaction time scores was significant only for IBMT group [t(17) = 6.949, *p* < 0.001]. Prior to training, the IBMT and relaxation groups did not differ in reaction times and accuracy scores (*P* > 0.05). After training, a *t*-test showed that the IBMT group demonstrated superior performance on the Stroop task, as indicated by significantly faster reaction times than relaxation group in the congruent [t(33) = 2.717, *p* < 0.05], the incongruent [t(33) = 3.745, *p* < 0.01], the neutral [t(33) = 2.632, *p* < 0.05] conditions, as well as smaller conflict scores [t(33) = 3.611, *p* < 0.01].

**FIGURE 1:**
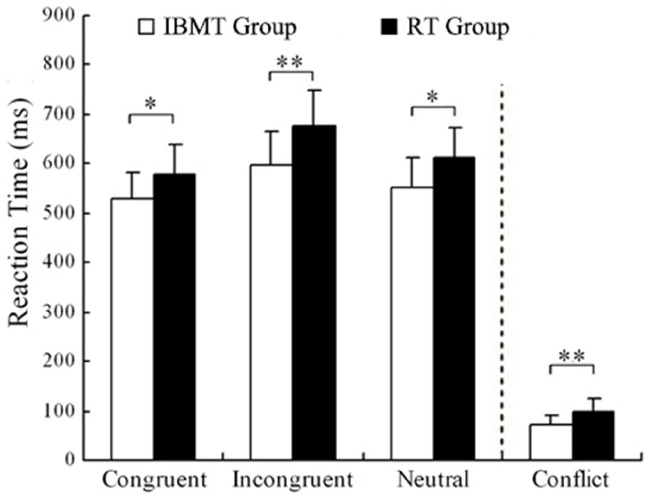
**Comparison of Reaction Time in Stroop task between two groups after training.** Conflict is the result of incongruent reaction time minus the congruent reaction time (*p < 0.05, **p < 0.01). IBMT, integrative body-mind training; RT, relaxation training.

For accuracy analysis (at pre-session, Congruent 97.5% vs. Incongruent 94.0% in IBMT and 97.0 vs. 93.9% in relaxation; at post-session, Congruent 97.9% vs. Incongruent 95.3% in IBMT and 97.8 vs. 95.5% in relaxation), no significant differences were found between two groups at each session, nor between two sessions in each group (all *p* > 0.05). This suggested that the superior performance on Stroop reaction times at post-training was not due to participants responding less carefully. Figure 1 shows a comparison of IBMT and relaxation groups in the Stroop task after training.

**FIGURE 2:**
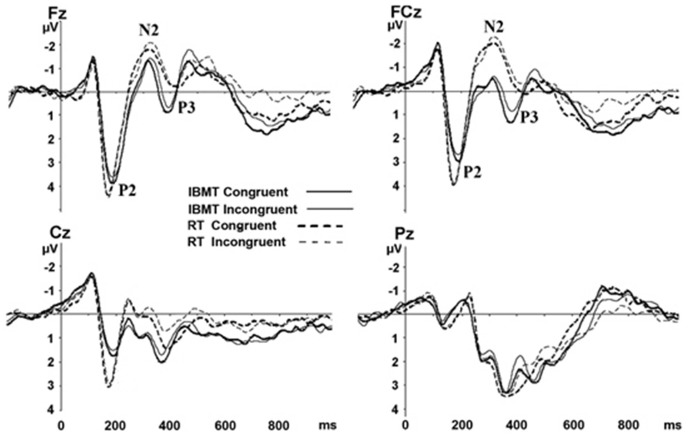
**Grand-average ERPs for congruent and incongruent conditions at Fz, FCz, Cz, and Pz between two groups after training.** The letters F, C, P, and O stand for frontal, central, and parietal lobes, respectively. A “Z” (0) refers to an electrode placed on the midline. IBMT, integrative body-mind training; RT, relaxation training.

To examine the training effects on brain activity, we then computed differences in ERP components between the two groups at the post-training session. Four midline sites along the anterior-posterior axis (Fz, FCz, Cz, and Pz) were selected based on previous research findings that showed frontopolar, frontocentral, central, and parietal scalp regions have Stroop-related changes (Liotti et al., 2000; Markela-Lerenc et al., 2004; Qiu et al., 2006). Grand-average ERPs waveforms for both groups at Fz, FCz, Cz, and Pz were shown in Figure 2. We included the Pz data in Figure 2 but restricted analysis to the anterior sites.

After training, the IBMT group showed significantly shorter P3 latency than the relaxation group at FCz in congruent [*F*(1,33) = 6.584, *p* < 0.05] and incongruent [*F*(1,33) = 10.605, *p* < 0.01] conditions; at Fz in congruent [*F*(1,33) = 6.881, *p* < 0.05] and incongruent [*F*(1,33) = 13.111, *p* < 0.01] conditions; and Cz [congruent: *F*(1,33) = 5.126, *p* < 0.05; incongruent: *F*(1,33) = 3.359, *p* = 0.079]. The P3 amplitudes were significantly larger in IBMT group than the relaxation group at FCz [congruent: *F*(1,33) = 15.102, *p* < 0.01; incongruent: *F*(1,33) = 16.365, *p* < 0.01], Fz [congruent: *F*(1,33) = 10.264, *p* < 0.01; incongruent: *F*(1,33) = 10.838, *p* < 0.01], and Cz [congruent: *F*(1,33) = 2.892, *p* = 0.101; incongruent: *F*(1,33) = 11.801, *p* < 0.01].

**FIGURE 3:**
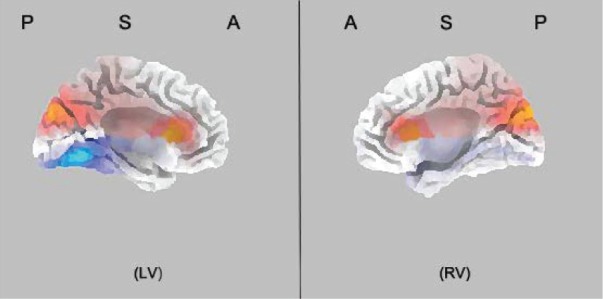
**sLORETA statistical non-parametric maps for the incongruent conditions for P3 ERP component.** P3 localized bilaterally at the dorsal part of the anterior cingulate cortex (ACC; BA 24/BA 32, *p* < 0.05). The time window is 400–500 ms. The letters P, S, A stand for posterior, superior, and anterior view of brain portion, respectively. LV, left view; RV, right view. The warm colors mean increased activity and cold colors represent decreased activity.

Source analysis using sLORETA showed that the P3 localized bilaterally to the dorsal part of the ACC (BA 24/BA 32, *p* < 0.05; Pascual-Marqui et al., 2002; Lorenzo-López et al., 2008; Figure 3). There was no significant difference between two groups for the N2 latency, and the effect is mainly observed in the amplitude of the component. The N2 amplitudes were smaller in IBMT group than the relaxation group at FCz [congruent: *F*(1,33) = 7.508, *p* < 0.05; incongruent: *F*(1,33) = 15.651, *p* < 0.01], Cz [congruent: *F*(1,33) = 7.270, *p* < 0.05; incongruent: *F*(1,33) = 13.248, *p* < 0.01], and Fz [congruent: *F*(1,33) = 3.521, *p* = 0.07; incongruent: *F*(1,33) = 5.709, *p* < 0.05]. Inspection of the topographical voltage map for N2 component in the incongruent condition indicated that group differences were mainly over frontal midline and left temporo-parietal scalp regions.

The IBMT group also showed significantly delayed P2 latency and smaller P2 amplitudes than relaxation group at frontal midline (FCz and Cz) and other brain regions (all *p* < 0.05).

## Discussion

Using the classic Stroop task, 5 h of IBMT improved the ability to resolve conflict compared to an active relaxation control. This result supported our previous findings using the attention network test to measure conflict resolution (Tang et al., 2007; Fan et al., 2014).

A series of behavioral and imaging studies have established that IBMT can improve attention and self-control after only 2 h of practice through increased activation in the ACC accompanied by improved connectivity to the parasympathetic system (Tang et al., 2007, 2009, 2010; Tang and Posner, 2009, 2014). These measures were obtained at rest and thus reflect a change in brain state (Tang et al., 2012b). This change in brain state is not achieved by RT which served as a control condition in these studies (Tang et al., 2009).

In the current study, we found significant differences between two groups in brain electrical activity over frontal midline ACC regions after training. The P3 amplitude is thought to reflect the amounts of attentional resources employed in a given task (Polich, 2007). Our study is consistent with the idea that meditation practice affects attentional resource allocation and increases the efficiency of conflict resolution (Cahn and Polich, 2006; Sarang and Telles, 2006; Slagter et al., 2007; Kozasa et al., 2012). The shorter P3 latency also agrees with the faster overall reaction time following IBMT.

These findings provide a likely account of how a change in brain state influences the ability to resolve conflict. Posterior brain potentials did not differ between two groups but the frontal midline N2, which has often been related to the effort to monitor conflict, was greatly reduced in the IBMT, especially in the more dorsal part of the ACC. Thus, the smaller N2 may suggest less effort needed to monitor conflict after meditation training and quicker resolution as shown by both reaction time and P3 latency. These results are consistent with our series of randomized studies that IBMT changes brain state and resolves conflict with less effort and more efficiency (Tang et al., 2007, 2012b; Tang and Posner, 2009, 2014; Xue et al., 2014).

P3 latency is proportional to stimulus evaluation time and individual differences for P3 latency are correlated with mental functions, such that shorter latencies are related to superior cognitive performance (Ilan and Polich, 1999; Rosenfeld and Skogsberg, 2006; Polich, 2007). The neuropsychological tests that produce the strongest correlation between P3 latency and cognitive capability assess how rapidly subjects can allocate attentional resources. In addition, P3 latency increases with normal aging and cognitive impairment (Polich, 1996; Neuhaus et al., 2007). In the current study, after training, IBMT decreased P3 latency at Fz and FCz compared to relaxation in both congruent and incongruent conditions. These findings may be consistent with increased attention, creativity and working memory in our studies, and may indicate that IBMT increases cognitive capability through the reorganization of attention resources in midline cortices (Tang et al., 2007; Ding et al., 2014a,b).

In a 16-week breathing meditation study using Stroop task (Moore et al., 2012), meditation group showed increased N2 (160–240 ms) component than a waiting list control group at left medial and lateral occipitotemporal areas, and decreased P3 (310–380 ms) component at right lateral occipitotemporal and inferior temporal areas respectively. These results involved the same components, but different direction and localization from what we found using IBMT. This raises the possibility that meditation type or length of practice may affect the brain processing involved in conflict resolution (Cahn and Polich, 2009; Tang and Posner, 2013). However, the use of a waiting list control could mean that factors other than the training were involved in the specific findings.

A recent fMRI study compared the performance of meditators and matched controls in the Stroop task. There were no differences in the Stroop task interference effect between the groups, but non-meditators showed greater activity than meditators in the right medial frontal, middle temporal, precentral and postcentral gyri and the lentiform nucleus during the incongruent conditions (Kozasa et al., 2012). Authors explained that less brain activity following meditation training indicated increased brain efficiency in the task. However, this could have been a property of those who chose meditation rather than being cause by training.

In sum, 5 h of IBMT induces a brain state that modulates the activity of ACC and improves information processing including the resolution of Stroop conflict. These findings are compatible with the idea that IBMT improves cognitive flexibility and reduces habitual response via enhanced self-control (Tang et al., 2015).

### Conflict of Interest Statement

The authors declare that the research was conducted in the absence of any commercial or financial relationships that could be construed as a potential conflict of interest.
